# Interstitial 12p Deletion Syndrome: Revised Minimal Critical Region and Review of the Literature

**DOI:** 10.3390/genes17010104

**Published:** 2026-01-19

**Authors:** Flavia Privitera, Stefano Pagano, Lorenzo Cipriano, Giulia Nutile, Annarita Milone, Filippo Maria Santorelli

**Affiliations:** 1 Department of Developmental Neuroscience, IRCCS Stella Maris Foundation, Via Dei Giacinti 2, 56128 Pisa, Italy; flavia.privitera@fsm.unipi.it (F.P.); stefano.pagano@fsm.unipi.it (S.P.); lorenzo.cipriano@fsm.unipi.it (L.C.);; 2 Molecular Medicine, IRCCS Fondazione Stella Maris, Via Dei Giacinti 2, 56128 Pisa, Italy; 3 Developmental Psychiatry and Psychopharmacology, IRCCS Stella Maris Foundation, 56128 Pisa, Italy

**Keywords:** 12p deletion syndrome, skeletal abnormalities, *CCDC91*, *DENN5B*

## Abstract

Background: Interstitial deletions of the short arm of chromosome 12 are rare, and very little is known about the potential genetic basis of the most common phenotypic presentations to date described in the literature. Methods: In the present study, we present a new patient carrying a heterozygous *de novo* 12p deletion, identified by a-CGH. Results: Comparison between the new case with phenotypically similar 12p-deleted patients drawn from the literature and from the DECIPHER (the DatabasE of Chromosomal Imbalances and Phenotypes using Ensembl Resources) database allowed us to analyze 22 cases and to define a revised minimal critical region not previously considered. Discussion: Within the new minimal critical region, we identified genes intolerant to haploinsufficiency, highlighting the involvement of *PTHLH* and *CCDC91* in the onset of skeletal abnormalities and proposing the involvement of *PPFIBP1* in neurodevelopmental disorders (although it has previously been associated only with autosomal recessive conditions). Conclusions: We suggest that clinical severity in cases with 12p deletions varies depending on the cytobands involved, being more moderate when they occur at 12p11—where the gene *DENND5B* (12p11.23) has recently been associated with a dominant neurodevelopmental disorder—than at 12p12.

## 1. Introduction

Interstitial deletions of the short arm of chromosome 12 are rare, with about 40 patients reported to date [1,2,3,4,5,6,7,8,9]. Based on their location, these deletions have been divided into four groups: (1) those occurring from p1→ p11; (2) those occurring from p13→ p11; (3) those confined to band 12p13; and (4) those extending to the terminal portion of 12p [1,2]. Common phenotypic findings associated with interstitial 12p deletions include intellectual disability, developmental delay, brachydactyly and clinodactyly, and microcephaly and/or craniofacial abnormalities [1,2,3]. Skeletal and dental abnormalities, as well as optic atrophy, are also commonly described [1,2,3,4,5,6,7,8,9]. Nevertheless, the phenotypic expression is highly variable, and the genes potentially correlated with specific clinical features remain largely unknown. One exception is the parathyroid hormone-like hormone gene (*PTHLH*, *168470), which is clearly associated with brachydactyly type E2 (MIM #613382), inherited in an autosomal dominant manner [4].

In this study, we present a new patient carrying a *de novo* 12p interstitial deletion, showing autism spectrum disorder (ASD), learning difficulties, psychomotor delay, and facial dysmorphisms. We then consider other similar deletions already reported in the literature and in DECIPHER (the DatabasE of Chromosomal Imbalances and Phenotypes using Ensembl Resources, https://www.deciphergenomics.org/, accessed on 1 June 2025), with the aim of summarizing the main recurrent clinical features, revising the minimal critical region, and proposing new candidate genes for some of the most recurrent traits associated with 12p deletions, highlighting differences between the cytobands involved.

## 2. Materials and Methods

### 2.1. Patient Recruitment

A blood sample from the new *de novo* patient carrying a 12p deletion was collected at our clinical laboratories—Molecular Medicine, IRCCS Fondazione Stella Maris (Pisa, Italy). In July 2025, we also invited the European Reference Network ITHACA (Intellectual disability, TeleHealth, Autism and Congenital Anomalies, https://ern-ithaca.eu/) to collaborate, but no other cases similar to our were reported Genetic counseling was provided, and data on the patient’s family history and clinical and dysmorphic features were collected. Informed consent for the study was obtained from the parents in accordance with the Declaration of Helsinki.

### 2.2. Genetic Tests

Genetic tests were performed on DNA isolated from a peripheral blood sample. High-resolution whole-genome array-based comparative genomic hybridization (aCGH), using SurePrint G3 Human CGH Microarray 8 × 60 k (Agilent Technologies, Santa Clara, CA, USA) with 41 Kb genome-wide median probe spacing, was carried out; also, whole-exome sequencing (WES), using a 150 bp paired-end protocol on a NovaSeq 6000 sequencer (Illumina Inc., San Diego, CA, USA) with 98% coverage of the total target > 20X, was performed. Reads were mapped against the hg19 reference genome using the Burrows–Wheeler aligner [10], and variants were called using HaplotypeCaller from the GATK suite v.4.0 (Broad Institute, Cambridge, MA, USA). Exome data were analyzed using enGenome-eVai v.3.6 (CE-IVD) and the Integrative Genomic Viewer (IGV; http://igv.org/), both accessed on 2 July 2025. All copy-number variants (CNVs) were analyzed and mapped using the Human Genome Assembly GRCh37/hg19, and then interpreted using the Database of Genome Variants (https://dgv.tcag.ca/dgv/app/home, accessed on 30 May 2025), DECIPHER (https://www.deciphergenomics.org, accessed on 1 June 2025), the UCSC genome browser (https://genome.ucsc.edu, accessed on 30 May 2025), the Database of Human CNVs (https://gvarianti.oasi.en.it/#, accessed on 30 May 2025), SFARI (Simon’s Foundation Autism Research Initiative, https://gene.sfari.org, accessed on 30 May 2025), and OMIM (Online Mendelian Inheritance in Man database, https://www.omim.org/, accessed on 30 May 2025).

In addition to the American College of Medical Genetics guidelines [11], various databases—the NCBI’s dbSNP (https://www.ncbi.nlm.nih.gov/snp/, accessed on 2 July 2025), ClinVar (https://www.ncbi.nlm.nih.gov/clinvar/, accessed on 2 July 2025), LOVD v.3.0 (Leiden Open Variant Database, https://www.lovd.nl/, accessed on 2 July 2025), Franklin by Genoox (https://franklin.genoox.com/clinical-db/home, accessed on 2 July 2025), Mobi Details (https://mobidetails.iurc.montp.inserm.fr/MD/, accessed on 2 July 2025), and VarSome (https://varsome.com/, accessed on 2 July 2025)—were consulted for the classification and interpretation of the variants detected by WES.

## 3. Results

### 3.1. Clinical Description and Genetic Findings

**Figure 1:**
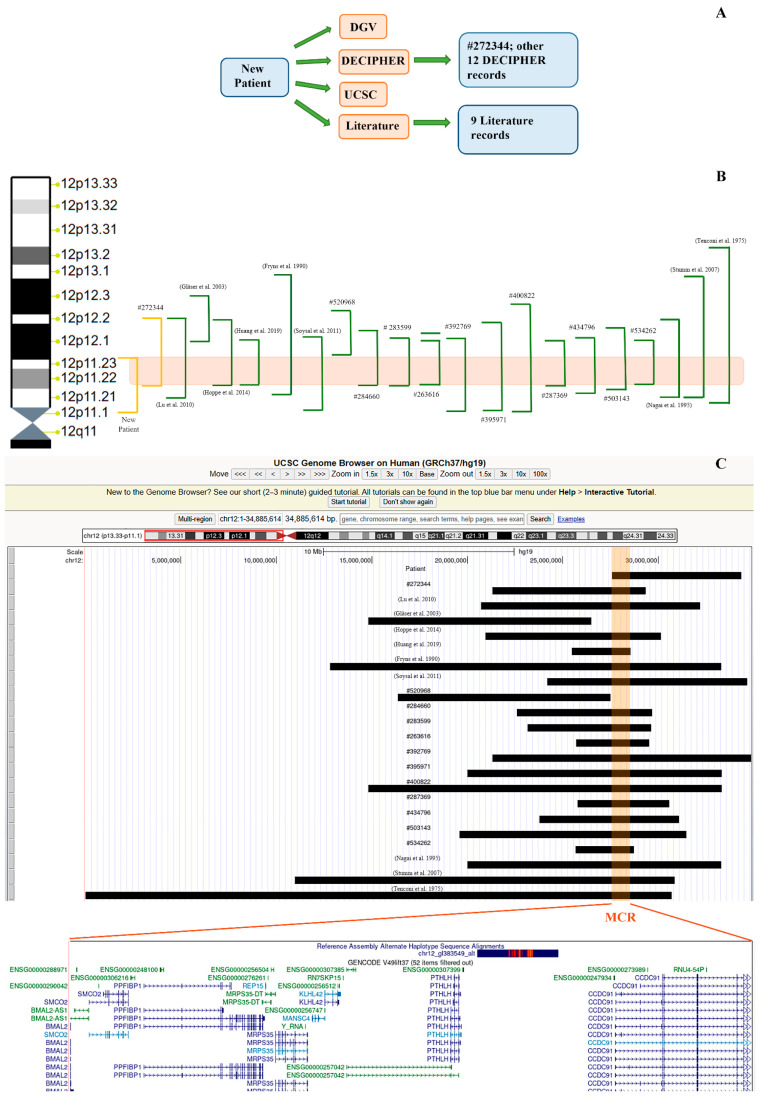
(**A**) Flowchart used to investigate the current study. From our new patient, and from consultation of the literature and interpretation databases (DGV, https://dgv.tcag.ca/; DECIPHER, https://www.deciphergenomics.org/; UCSC, https://genome.ucsc.edu/; PubMed, https://pubmed.ncbi.nlm.nih.gov/), we found the DECIPHER record #272344, of which we obtained detailed clinical information; then, other records and other cases from the literature were used to further investigate the genotype–phenotype correlation of 12p interstitial deletions (all the DECIPHER records are indicated with #). (**B**) Overview of deleted regions in the case series. On the left: ideogram of 12p, from the terminal cytoband 12p13.33 to the centromeric one 12p11.1 (plus cytoband 12q11). On the right: new patient and record #272344 indicated in yellow; patients from the literature and from DECIPHER indicated in green [1,2,3,4,5,6,7,8,9]. (**C**) Overlapping of 12p deletions seen by UCSC Genome Browser (https://genome.ucsc.edu/) and focus on the genes involved in the Minimal Critical Region (MCR). Except for the case reported by Gläser B. et al. [2] and patient #520968, deletions are predominantly located between cytobands 12p12.1 and 12p11.1 [1,2,3,4,5,6,7,8,9]: analysis of overlap between the deleted remaining regions showed a minimal critical region covering 809.94 Kb (orange bar) and involving cytobands 12p11.23 and 12p11.22, with genomic coordinates starting at 27,573,443 and ending at 28,536,312 (breakpoints referring to the Human Genome Assembly GRCh37/hg19).

The patient we present is a 15-year-old boy, the only child born to unrelated parents. His mother exhibits anxious personality traits, while his father has a history of panic attacks, treated with escitalopram and psychotherapy. The patient was born via vaginal delivery at term following a normal pregnancy; his birth weight was 3.78 kg (50–75th percentile). He babbled and walked independently at 12 months. Neuropsychiatry assessment following the emergence of impulsive behavior, motor and vocal tics, difficulty relating to peers, attention deficit, and learning disability disorder resulted in diagnoses of anxiety disorder, phobias and obsessive–compulsive traits, ASD, and attention deficit hyperactivity disorder (ADHD). Cognitive functioning was characterized by a non-homogeneous profile. Scores for verbal comprehension and for visual-perceptual reasoning and processing speed were in the normal range, whereas working memory was borderline. Electroencephalogram (EEG) showed sporadic, independent sharp wave activity over the posterior vertex/parieto-occipital and fronto-centrotemporal regions; brain magnetic resonance imaging (MRI) and echocardiogram were normal. On physical examination, the following were recorded: weight 42 kg (45th percentile), height 150.5 cm (30th percentile), and occipital frontal circumference (OFC) 55 cm (39th percentile). The patient presented with frontal bossing, bitemporal constriction, hypertelorism, down-slanting palpebral fissures, a saddle nasal bridge, dental malalignment, bilateral brachydactyly, moderate scoliosis, and bilateral flat foot. In this patient, aCGH showed a 6.77 Mb heterozygous 12p deletion, arr[GRCh37] 12p11.23p11.1(27,573,443_34,345,585)x1 (Figure 1, Supplementary Table S1). Segregation analysis of the parents confirmed its *de novo* origin. WES analysis, performed to exclude other significant genetic causes not detectable by aCGH, was negative.

Based on these clinical and molecular data, and through a literature review and a consultation of DECIPHER and other interpretation databases (Figure 1A), we identified 21 additional cases with deleted regions fitting the one identified in the new patient (Figure 1B,C). The research focused on the most commonly described phenotypic traits, as well as on the overlap of 12p deletions found by genetic tests. Clinical findings of the entire case series are presented in Table 1 and Table 2. In particular, thanks to the “Contact” function of DECIPHER, we obtained detailed information about a DECIPHER record (#272344, Figure 1A–C), allowing us to develop the present study and highlight differences among 12p deletions.

### 3.2. Summary of Clinical Findings in 12p Interstitial Deletions

Overall, digital anomalies and facial dysmorphisms were found to be common characteristics in our case series of 12p interstitial deletions, occurring in 82% and 77%, respectively (18/22 and 17/22, Table 1 and Table 2). Psychomotor delay and optic atrophy were features in 50% (11/22), skeletal abnormalities in 45% (10/22), and microcephaly and dental abnormalities in 32% (7/22). Intellectual disability (32%, 7/22), speech delay (23%, 5/22), and cardiovascular anomalies (18%, 4/22) were less frequent, while movement disorders (14%, 3/22), ASD, and abnormal brain MRI and EEG (9%, 2/22) were reported in very few cases, although we note that the selected case series includes a fetus and newborns for whom no clinical follow-up is available. ADHD was reported only once (4.5%, 1/22). We further note that phenotype descriptions in DECIPHER are restricted to Human Phenotype Ontogeny (HPO) terms, being oversimplified or incomplete and potentially affecting the reliability of our analysis. Other clinical features, observed in some patients, include anxiety disorder; genital hypoplasia or cryptorchidism; Turner-like stigmata; splenomegaly; horseshoe kidney; cutaneous involvement; ureteral duplication; iron deficiency anemia; hypothyroidism; elevated circulating creatine kinase concentration; and type I diabetes mellitus (Table 1 and Table 2).

In our new patient and in case #272344, digital and skeletal anomalies, facial dysmorphisms and psychomotor delay were confirmed as recurrent phenotypic traits; the new patient showed a milder phenotype than #272344, which more oriented towards neuropsychiatric features, including impulsive behavior, anxiety disorder, phobias and obsessive–compulsive traits, ASD, and attention deficit hyperactivity disorder (ADHD)—traits not frequently reported in 12p deletions. Case #272344 instead presents the features frequently described in the literature, including ophthalmological anomalies, microcephaly, and dental irregularity; no updated information on possible neuropsychiatric features is available, since no clinical follow-up has been scheduled over time for this case. Interestingly, both patients presented EEG anomalies, which are not commonly reported.

### 3.3. Genetic Findings

Full genetic findings are detailed in Supplementary Table S1. CNVs were interpreted using the databases listed in Section 2.2. Deletions in the literature were considered according to the involved cytobands and described phenotypes. Patients listed in DECIPHER were detected using the “Matching Patient Variant” function, considering only “Copy-number variants” and excluding “Sequence variants” and “Other variants”, since we were interested, for clinical interpretation, only in heterozygous overlapping deletions. Those inherited from parents or smaller than 2 Megabases (Mb), due to the significantly different number of genes involved, or whose deletions involved non-disease-associated genes, were not included in the study. Human Phenotype Ontology (HPO) was used to match patients in DECIPHER in order to find similar overlapping phenotypes (HPO codes are listed in Supplementary Table S2).

### 3.4. Minimal Critical Region on Chromosome 12p and Involved Genes; Statistical Analysis

Analysis of overlap between the deleted regions in the selected case series showed a Minimal Critical Region (MCR) covering 809.94 Kb and involving cytobands 12p11.23 and 12p11.22, with genomic coordinates starting at 27,573,443 and ending at 28,536,312 (Figure 1B,C). The involved genes are listed in Table 3, and they were evaluated for probability of loss-of-function intolerance (pLI) [12,13], predicted probability of haploinsufficiency (pHaplo) [13], loss-of-function observed/expected upper bound fraction (LOEUF) [13], ClinGen dosage sensitivity rates (https://clinicalgenome.org/), and expression pattern by the Genotype-Tissue Expression (GTEx) portal (https://gtexportal.org/).

**Figure 2:**
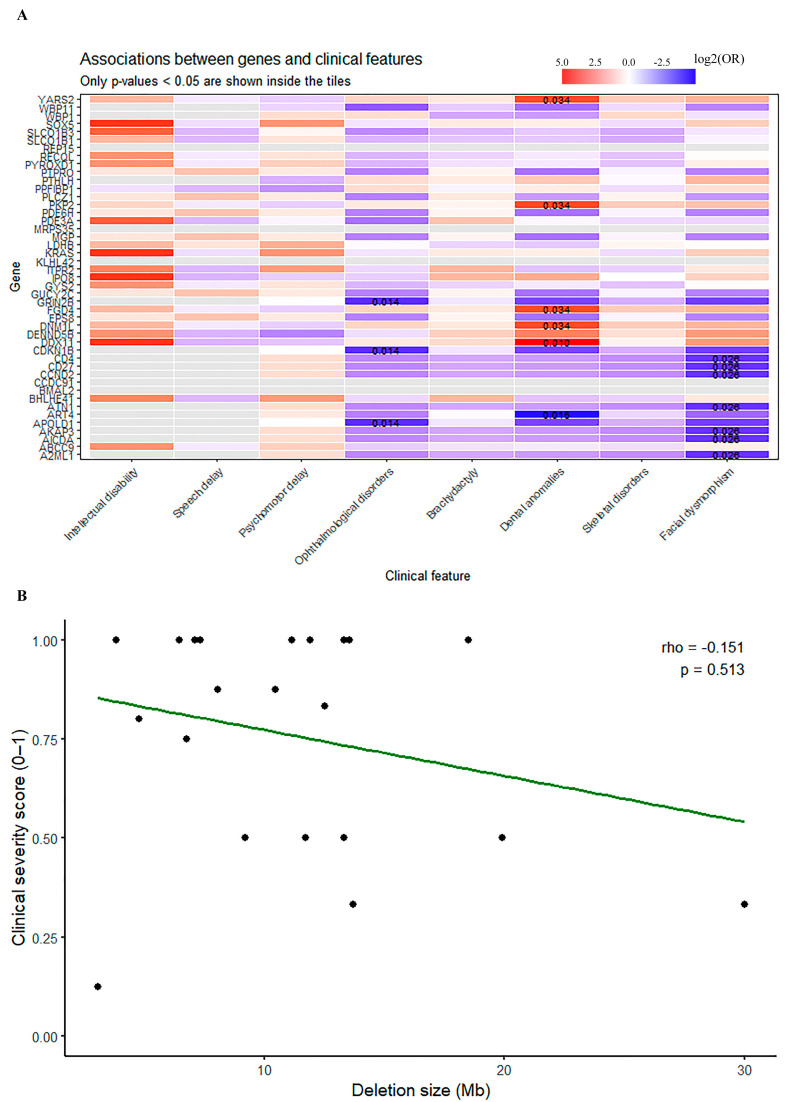
(**A**). Heatmap represents the association between genes and clinical features according to log2(OR) values. Red: positive association; blue: negative association; white: no relevant association. On the vertical axis, the list of analyzed genes (disease- and non-disease-causing, the latter limited to those present in the MCR to better study their pathogenic role); on the horizontal axis, the main clinical features observed in 12p interstitial deletions. Most relevant *p*-values (<0.05) are reported inside tiles. The more red a gene tends to be, the greater its association with the phenotype; the more it tends toward being blue, the more it will deviate from it. (**B**). Spearman’s rank order correlation to test the association between deletion size (Mb, horizontal axis) and the Clinical Severity Score (from 0 to 1, vertical axis). This association was not found to be significant.

For each gene involved in the deletions, both disease-causing and non-disease-causing (the latter limited to those present in the MCR only, to better study their pathogenic role), we evaluated its association with eight clinical features by calculating odds ratios (ORs) using 2 × 2 contingency tables. Gene status and clinical variables were coded as binary values (1 = present, 0 = absent). Only complete cases were included in each comparison. Because some contingency tables contained zero counts, we applied Haldane’s correction by adding 0.5 to all cells to ensure valid OR estimation. Odds ratios and 95% Wald confidence intervals were computed using the epitools package in R. Statistical significance was assessed using Fisher’s exact test. All analyses were performed independently for each gene–phenotype pair. A Clinical Severity Score (0–1) was generated to quantify overall phenotypic burden. Eight clinical features consistently reported across both the literature and DECIPHER cases were included: facial dysmorphisms, brachydactyly/hand–foot anomalies, psychomotor delay, speech delay, intellectual disability, optic atrophy/ophthalmological abnormalities, skeletal anomalies, and dental anomalies. Each feature was coded as present (1) or absent (0), while missing data were excluded. The score was defined as the proportion of present features among those assessed for each patient. A heatmap was then generated to appreciate the association between genes and clinical features (Figure 2A).

To investigate whether the extent of the 12p deletions contributed to phenotypic severity, we tested for an association between deletion size (Mb) and the Clinical Severity Score using Spearman’s rank order correlation (Figure 2B).

## 4. Discussion

Interstitial deletions of the short arm of chromosome 12 are rare, and very little is known about the potential genetic basis of the most common phenotypic traits thus far highlighted in the literature. We described a new *de novo* case carrying a 12p deletion and compared our findings with cases listed in the DECIPHER database, in particular with case #272344, for whom we had retrieved more detailed information. Other patients already described in the literature were also reviewed.

Facial dysmorphisms and brachydactyly are the main clinical features associated with 12p deletions (Table 1 and Table 2); these are followed by characteristics such as psychomotor delay, optic atrophy, intellectual disability, and skeletal abnormalities. Moreover, analysis of the overlap between the deleted regions observed in our 22-strong case series allowed us to revise the Minimal Critical Region. The revised region spans 809.94 Kb (Figure 1B,C) and includes the genes reported in Table 3. We then evaluated their association with eight clinical features by calculating odds ratios (ORs) using 2 × 2 contingency tables, calculated a Clinical Severity Score (0–1) to quantify the overall phenotypic burden, and tested for possible association between deletion sizes (Mb) and the Clinical Severity Score using Spearman’s rank order correlation.

Among them, *PTHLH*, known to be associated with brachydactyly type E2 (613382) and probably responsible for this phenotypic trait, is the gene most intolerant to haploinsufficiency. Even if mainly expressed in the mammary gland (Table 3), *PTHLH* plays a critical role in bone development, being involved in chondrocyte proliferation through its receptor PTHRP [14]. The effects of PTHRP on chondrocyte differentiation are also mediated via the regulation of the phosphorylation of SRY-box transcription factor 9 (Sox9), a transcription factor important for chondrocyte differentiation, and by suppressing the Runt-Related Transcription Factor 2 (Runx2), a transcription factor essential for osteoblast differentiation [14]. Interestingly, long fingers but no brachydactyly were observed in case #272344, although *PTHLH* was included in the 12p deletion in this patient. The heatmap in Figure 2B does not actually support a strong association with brachydactyly—although it is still included among the genes with OD > 0 (Figure 2A), as it seems to be positively associated with dental anomalies, dysmorphic features, and ophthalmological disorders, slightly.

The second gene worthy of note was the coiled-coil domain containing 91, *CCDC91* (*617366). Although not yet associated with disease in OMIM and apparently highly expressed in the testis (Table 3), recent studies have linked this gene to Diffuse Idiopathic Skeletal Hyperostosis (DISH, #106400) [15] and Ossification of the Posterior Longitudinal Ligament (OPLL, 602475). It has also been recently associated with autosomal dominant acrokeratoelastoidosis, using a short hairpin RNA (shRNA) knockout model in human skin fibroblast and CRISPR/Cas9 knockout in HEK293T cells to assess gene functions in the progression of elastic fiber biosynthesis [16]. *CCDC91* is a known target of Runx2 [17], and variants affecting its expression have been implicated in musculoskeletal traits such as height, estimated bone mineral density, spine bone area, neck bone area, OPLL [18], osteoporosis [19], and osteoarthritis [20]. Therefore, its possible involvement in the onset of skeletal abnormalities cannot be excluded.

Another interesting gene falling within the revised minimal critical region is PPFIB scaffold protein 1 (*PPFIBP1*, *603141). This gene, which is ubiquitously expressed in tissues (Table 3), is associated with neurodevelopmental disorder with seizures, microcephaly, and brain abnormalities (#620024). It encodes a protein termed liprin-β1, capable of heterodimerizing with the homologous liprin-α proteins [21]—major scaffold proteins that, by forming large complexes, contribute to synapse formation, synaptic signaling, and axonal transport. It has also been suggested that liprin-β1 may play a role in the regulation of liprin-α-mediated protein assemblies, which are essential for neurodevelopment [21]. Moreover, automated behavioral phenotyping of a Caenorhabditis elegans PPFIBP1/hlb-1 knockout model revealed defects in spontaneous and light-induced behavior, confirmed resistance to the acetylcholinesterase inhibitor aldicarb, and suggested a defect in the neuronal presynaptic zone [21]. Although *PPFIBP1* has a pLI of 0 and a pHaplo of 0.81 (Table 3)—values consistent with its autosomal recessive disease mechanism— and has a OD < 0 for intellectual disability in the heatmap (Figure 2A), a role in the psychomotor deficit observed in 12p-deleted patients cannot be entirely excluded, particularly in the context of a multigene deletion syndrome. An identified microdeletion lying within the revised minimal critical region, containing only *PTHLH* and *PPFIBP1* as disease genes, was described in a family with brachydactyly and learning disabilities, supporting the possible involvement of *PPFIBP1* in the neurodevelopmental phenotype [13]. Patients #263616 and #287369, both carriers of deletions encompassing a number of disease genes including *PPFIBP1*, showed intellectual disability and global psychomotor delay (Table 2), while no neurodevelopmental disorders were apparent in the family described by Huang et al. [4], or in patient #534262 (Table 1 and Table 2). However, as previously mentioned, phenotypes reported in DECIPHER may not comprehensively reflect patients’ actual clinical presentations.

The other genes included in the MCR encoded proteins involved in different cellular pathways and were ubiquitously expressed, except for RAB15 Effector Protein (*REP15,* *610848) gene, which seems to be well represented in the digestive tract (Table 3). They have not yet been associated with diseases, and very little is known about knockout studies on them. Regarding the basic helix–loop–helix ARNT like 2 (*BMAL*, *614517) gene, it has been highlighted that deletion in mice leads to increased body weight gain during diet-induced obesity, triggering the inflammatory response, reducing lipid storage capacity and increasing hepatic storage and insulin resistance in the liver [22]. Figure 2A shows that *CCDC91*, *REP15*, *BMAL*, the mitochondrial ribosomal protein S35 (*MRPS35*, *611995) gene, and the Kelch like family member 42 (*KLHL42*, *618919) gene appear to have OD = 0 for the main phenotypes investigated; this is understandable, since little is still known about their association with pathological phenotypes, and further studies are needed to better define their possible clinical effect.

Overall, Figure 2A shows that there are relatively few significant associations in the context of the entire matrix. However, some clinical features show more evidence of associations than others, for instance, dental anomalies. It seems that genes such as the Tyrosyl-tRNA synthetase, mitochondrial MT-TYRRS *YARS2* (*610957), the Fyve, RhoGEF and PH-Domain-Containing Protein 4 *FGD4* (*611104), the Dynamin 1-Like *DNM1L* (*603850) and the Dead/H-Box Helicase 11 *DDX11* (*601150) are statistically significant in correlation with this phenotype, even though these genes are associated with conditions that would explain something completely different (#613561; #619311; #610708; #613398, respectively). Individual expression pathways should be investigated to assess shared features.

Depending on the size of the deleted region, psychomotor development could be due to the loss of function of more than one gene. As shown by Figure 1B, C, most of the deletions seem to occur between cytoband 12p12.1 and cytoband 12p11.1; larger deletions are very rare and are expected to lead to more complex phenotypes, as was observed in #272344. In this patient, the phenotypic contribution of the Xq27.1q27.3 duplication involving SRY-BOX3 (*SOX3*, *313430) could not be further excluded, since duplications encompassing this gene are associated with X-linked hypopituitarism and intellectual disability [23]. It is likely that the small pituitary gland shown in case #272344 may have also been due to this chromosomal aberration; the other genes involved in Xq27.1q27.3 duplication have not been reported to date in association with pathological phenotypes, except for the ATPase, Class VI, Type11C (*ATP11C*, *300516) gene, which is entirely duplicated but only associated with X-linked recessive hemolytic anemia (#301015).

Regarding the correlation between the deletion size and the clinical severity, Spearman’s rank order correlation did not yield significant results in our case series. This may be due to several factors: (1) clinical severity is not a linear and continuous variable, as it is often classified into categories (“mild”, “moderate”, and “severe”), rather than represented with ordinal values; (2) the phenotype may be the result of individual variability and genetic/epigenetic background; (3) not all genes in the deletions may contribute equally. Finally, the case series in analysis may also be too small to detect a positive correlation.

In accordance with the hypothesis that not all the genes contribute with the same importance to the phenotype, we have to consider that cytoband 12p12.1 includes the SRY-box transcription factor 5 (*SOX5*, *604975) gene, already known to be associated with Lamb–Shaffer syndrome (#616803). This syndrome recapitulates all the clinical features mentioned in our summary of clinical findings [6,24]; however, 12p deletion phenotypes cannot be attributed to *SOX5* alone, since similar clinical presentations have been reported in deletions that do not include this gene. Another interesting new gene from a neurodevelopmental perspective is DENN domain containing 5B (*DENND5B*, *617279, cytoband 12p11.21): this gene, positively associated with intellectual disability, dental anomalies and facial dysmorphism in Figure 2A, encodes a member of the DENND5 protein subfamily that seems to contribute to synaptic plasticity through the modulation of synaptic vesicles, axonal trafficking, and neurotransmitter release [25]. Heterozygous *de novo* variants in *DENND5B* with predicted loss of function have been associated with neurodevelopmental disorders with dysmorphic features and behavioral dysregulation (the latter including ASD, ADHD, and anxiety disorder) [25]. Altogether, this suggests that deletions involving the cytoband 12p11 may be associated with milder neurodevelopmental phenotypes than the syndromic forms involving the cytoband 12p12.1—a suggestion consistent with the findings on our new patient and case #272344, which showed the latter to have the more severe phenotype.

In conclusion, the present study set out to further delineate the genotype–phenotype correlation associated with 12p deletion and propose a minimal critical common region including the candidate genes *PTHLH*, *CCDC91*, and *PPFIBP1*. However, we recognize obvious limitations. In particular, functional data or transcriptomic investigations should be considered in order to understand the role of *CCDC91* and its involvement in osteogenesis and skeletal abnormalities, and the role of heterozygous deletions in *PPFIBP1* in the onset of impaired neurodevelopmental phenotypes. Furthermore, databases such as DECIPHER allow us to easily overlap chromosomal aberrations and compare phenotypes, but these ones are often oversimplified and can be misleading. Scientific collaborations between specialized centers are always strongly recommended to overcome some of these issues. Further studies of genes not yet associated with disease included in the MCR are also necessary to understand their potential role in causing other frequent features of the 12p deletion syndrome such as optic atrophy. Finally, additional patients harboring large deletions in cytoband 12p11 should be investigated to clarify the neuropsychiatric phenotypes associated with these regions.

## Figures and Tables

**Table 1:** Clinical findings from the patient described in the present study and those drawn from the literature.

Clinical Features	New Patient	Hong-Yong L. et al. [1]	Glaser B. et al. [2]	Hoppe A. et al. [3]	Huang J. et al. [4]	Fryns JP. et al. [5]	Soysal Y. et al. [6]	Nagai T. et al. [7]	Stumm M. et al. [8]	Tenconi R. et al. [9]
**Sex; age**	M; 15 y.o.	F; 13 y.o.	M; 6 months	M; 8 months	Family of nine affected individuals	F; 7.5 months	F; 12 y.o.	M; 5 y.o.	F; fetus	M; 2 months
**Facial dysmorphism**	+: frontal bossing; bitemporal constriction; hypertelorism; downslanting palpebral fissures; saddle nasal bridge; bilateral flat foot.	+; short stature; round face	+; unilateral cleft lip; bilateral cleft palate; slight facial asymmetry, low-set and dysplastic ears; large and flat nasal bridge; short palpebral fissures with bilateral epicanthic folds; short neck and a broad thorax with wide-set mamillae.	+; small ears; epicanthus; broad nasal bridge; and hypoplastic nostrils; inverted nipples; micropenis; hemangioma on his lower extremities; trunk hypotonia.	-	-	+; hypertelorism; downslanting palpebral fissures; mild inner epicantal folds; arched eyebrows; broad nasal base; bulbous nose; short philtrum.	+; craniofacial dysmorphisms	+	-
**ASD**	+	-	n.a.	n.a.	-	n.a.	+	-	n.a.	n.a.
**Intellectual disability**	-	+; moderate	n.a.	n.a.	-	n.a.	+; mild	+	n.a.	n.a.
**Speech delay**	+; moderate	-	n.a.	n.a.		n.a.	-	-	n.a.	n.a.
**ADHD**	+	-	n.a.	n.a.	-	n.a.	-	-	n.a.	n.a.
**EEG abnormalities**	+; sporadic, independent sharp abnormalities on the posterior vertex/parieto-occipital and fronto-centro-temporal region.	-	-	-	-	-	-	-	-	-
**Psychomotor delay**	-	-	+	+; significant.	-	+	+	-	-	+
**Movement disorder**	-	-	-	-	-	+	+	-	-	+
**Brain MRI alterations**	-	-	-	+	-	-	-	-	-	-
**Microcephaly/macrocephaly/abnormalities of the head**	-	-	+; microbrachycephaly.	+; postnatal microcephaly (−2.31 SD).	-	+; craniofacial dysmorphism.	+	-	-	+; craniofacial dysmorphism
**Atrophy of optic nerve/Ophthalmological problems**	+; hypertelorism.	-	+; unable to follow moving objects; no clear fixation. In the fundus of the right eye, partial pallor and vascular defects.	+; at 4 weeks of age, eye movement disorder of intermittent exotropia and discrete anisocoria. At 8 months, bilateral optic atrophy.	-	-	+; strabismus, myopia	-	-	-
**Brachydactyly or abnormalities of hand/feet**	+; bilateral.	+; characteristic brachydactyly, with the fifth toe overlapping the fourth one. Roentgenograms of hands and feet disclosed the bilateral brachydactyly, with shortened metacarpals of digits 3–5,middle phalanges of digit 5, and metatarsals of digits 4 and 5.	+; hands showed deeply furrowed single transverse creases and at the right foot the third toe overlaps the fourth one.	-	+; bilateral severe generalized brachydactyly of hands and feet and pectus carinatum.	+	+; phalangeal deformity in distal phalanges of hands; 5th finger camptodactyly; brachydactyly of the feet.	+	+	+
**Tooth abnormalities**	+; misalignment of teeth.	+; chaotic tooth arrangement.	-	-	-	-	+; irregular tooth arrangement.	+	-	-
**Cardiovascular anomalies**	-	-	+; hemodynamically insignificant small muscular ventricular septal defect and an atrial septal defect secundum.	-	-	+	-	-	-	+
**Skeletal abnormalities**	+; moderate scoliosis.	-	+; short tubular bones at ultrasound.	-	-	-	+; history of joint hypermobility; scoliosis; micro/retrognathia.	-	+; skeletal anomalies; marked micrognathia	-
**Other**	+; anxiety disorder; motor and sound tics.		+; pregnancy complicated at 28 weeks, when ultrasound diagnosed cleft lip and palate. At post-natal examination, horseshoe kidney.	+; splenomegaly	-	+; genital hypoplasia; Turner-like stigmata.		+; arterial hypertension	+; increased nuchal translucency at pregnancy	-

Abbreviations: +: present; -: absent. F: female; M: male. y: years; m: months; n.a.: not available; ASD: autism spectrum disorder; ADHD: attention deficit hyperactivity disorder. EEG: electroencephalogram; MRI: magnetic resonance imaging; SD: standard deviation.

**Table 2:** Clinical features from the DECIPHER patients.

Clinical Features	#272344	#520968	#284660	#283599	#263616	#392769	#395971	#400822	#287369	#434796	#503143	#534262
**Sex; age**	F; 15 y.o.	F; n.a.	M; 9 y.o.	M; 3 y.o.	M; 20 y.o.	F; 17 y.o.	M; 5 y.o.	F; 13 y.o.	M; 6 y.o.	M; 11 months	F; n.a.	F; 11 y.o.
**Facial dysmorphism**	+; long face with bitemporal narrowing; narrow palpebral fissures; long eyelashes; malar flattening; broad nasal bridge and tip with thick *alae naesi* and columella; long and smooth philtrum; small and receding chin; high and narrow palate with prominent palatal ridges.	n.a.	+; broad forehead.	+; short stature.	+; proportionate short stature.	+; abnormal pinna morphology; low-set ears; macrotia; protruding ears; stenosis of the external auditory canal; proportionate short stature; short and broad neck; bulbous nose; downslanted palpebral fissures; enlarged naris; exaggerated cupid’s bow; high palate; narrow forehead; narrow palate; prominent nasal bridge; highly arched eyebrow; frontal bossing.	+; short stature; low- set ears; depressed nasal bridge; round face; short nose; pectus excavatum; narrow chest; short thorax.	+; short stature; prominent nose.	+	+; short stature.	n.a.	+; short stature.
**Microcephaly/macrocephaly/abnormalities of the head**	+; -4SD	n.a.	n.a.	n.a.	n.a.	+	n.a.	+	+; trigonocephaly.	n.a.	n.a.	n.a.
**Intellectual disability**	-	n.a.	n.a.	n.a.	+	+	+	n.a.	n.a.	+; mild.	n.a.	n.a.
**Speech delay**	+	n.a.	+; oral apraxia	n.a.	n.a.	n.a.	+	+; dysarthria.	n.a.	n.a.	n.a.	n.a.
**Psychomotor delay**	+; moderate.	n.a.	+; mild.	+; global.	n.a.	+; significant.	n.a.	-	+; global.	+; motor delay.	n.a.	n.a.
**Atrophy of optic nerve/Ophthalmological problems**	+; divergent strabism, astigmatism and anisometropia; bilateral optic nerve hypoplasia and thin retrochiasmatic optical tracts.	n.a.	+; strabismus, optic nerve hypoplasia.	+; optic atrophy.	+; optic atrophy.	+; optic atrophy.	n.a.	+; hypertelorism; strabismus.	n.a.	+; myopia; optic nerve hypoplasia.	n.a.	n.a.
**Brachydactyly or abnormalities of hand/feet**	+; long fingers with broad thumbs and mild swelling of the distal interphalangeal joints, short toes with broad hallux and nail hypoplasia.	+; bilateral.	+; short 4th and 5th metacarpal; short distal phalanx of the thumb.	+; short digits.	+; short palm.	+; finger clinodactyly; genu valgum; short metacarpal.	+; cone- shaped epiphyses of the phalanges of the hand; finger clinodactyly; short phalanx of finger; short metacarpal; short foot.	n.a.	+; type E brachydactyly.	n.a.	n.a.	+; short 3rd, 4th, 5th metacarpal; short metatarsal.
**Tooth abnormalities**	+; conical shaped teeth with absent upper incisives after shedding of “double teeth”.	n.a.	n.a.	n.a.	n.a.	+; tooth malposition.	+; oligodontia.	n.a.	n.a.	n.a.	n.a.	+; delayed eruption of permanent teeth.
**Skeletal abnormalities**	+; kyphosis and thoracolumbar gibbus, and symptomatic spinal stenosis. An X-ray and spinal MRI showed abnormal vertebrae T12-L (wedging and irregular plates) and spinal stenosis at the L4-L5 level.	n.a.	n.a.	+; micrognatia.	n.a.	+; pneumatization of cranial sinuses; craniosynostosis; micrognathia; scoliosis.	+; coxa valga; narrow greater sciatic notch; abnormal pubic bone. morphology; osteochondroma.	+; hyperlordosis; micrognathia; scoliosis.	n.a.	n.a.	n.a.	+; cone- shaped epiphysis.
**Other**	+; small pituitary gland; episodes of low-frequency activity bilaterally on the posterior hemispheres.	n.a.	+; cryptorchidism.			+; hydrocephalus; ureteral duplication.	+; abnormality of the skin; aplasia/hypoplasia of the skin.	+; atrial septal defect; pulmonic stenosis; atopic dermatitis; anomalous pulmonary venous return.		+; iron deficiency anemia; hypothyroidism; elevated circulating creatine kinase concentration; type I diabetes mellitus.	n.a.	n.a.

Abbreviations “+”: present; n.a.: not available. F: female; M: male; y: years; m: months. Patients #520968 and #503143 were recruited based on their genetic findings, since their phenotypes were not shared.

**Table 3:** Gene mapping in the 12p11.23–p11.22 deleted region, listed by intolerance to haploinsufficiency.

Gene Full Name and Symbol (*OMIM)	Phenotype MIM Number (#)	Biological Activity	pLI ≥ 0	LOEUF~0	pHaplo ≥ 0.86	ClinGen Dosage Sensitivity Rate	**Main Expression Pattern (GTEx)**	**Association with Neurodevelopment Disorder**
*PTHLH* (*168470) Parathyroid hormone-like hormone	Brachydactyly, type E2 (#613382).	Encodes a parathyroid hormone-related protein (PTHrP) that is involved in the regulation of endochondral bone development.	0.93	0.56	0.92	Haploinsufficiency: 3; Triplosensitivity: 1	Breast mammary tissue; low expression in all the other tissues	No
*CCDC91* (*617366) Coiled-coil domain containing 91	-	Encodes for a critical accessory protein that promotes transport of carrier vesicles between the Golgi and lysosomes and lysosomal enzyme maturation.	0.06	1.08	0.09	-	Testis; low expression in all the other tissues	No
*REP15* (*610848) RAB15 Effector Protein	-	REP15 is a binding partner of the RAB GTPase family member RAB15 that facilitates transferrin receptor recycling from the endocytic recycling compartment.	0.02	1.87	0.11	-	Colon, sigmoid and transverse; small intestine; spleen; stomach; low expression in all the other tissues	No
*MRPS35* (*611995) Mitochondrial ribosomal protein S35	-	MRPS35 is a component of the small subunit of the mitochondrial ribosome that is encoded by the nuclear genome.	0.00	1.17	0.31	-	Ubiquitous	No
*BMAL2* (*614517) Basic helix–loop–helix ARNT like 2	-	BMAL2 is a basic helix–loop–helix (bHLH)/PAS domain transcription factor with a role in regulation of circadian rhythm.	0.00	0.83	-	-	-	No
*KLHL42* (*618919) Kelch like family member 42	-	KLHL42 is a cullun-3-interacting protein that functions as a CUL3 substrate adaptor.	0.00	1.32	0.48	-	Ubiquitous	No
*PPFIBP1* (*603141) PPFIB scaffold protein 1	Neurodevelopmental disorder with seizures, microcephaly, and brain abnormalities (620024).	*PPFIBP1* encodes for the liprin-beta1 protein, which has been shown to play a role in neuronal outgrowth and synapse formation in Drosophila Melanogaster.	0.00	0.91	0.81	-	Ubiquitous	Yes

-: not evaluated. pLI: Probability of loss-of-function intolerance. This is the probability that a gene is intolerant to loss-of-function (LOF) mutation based on the gene’s observed depletion of LOF variants in the Genome Aggregation Database (gnomAD); values range from 0 to 1, and the closer they are to one, the more they are intolerant to mutations. LOEUF: Loss-of-function observed/expected upper bound fraction. It is a quantitative measure of the observed depletion (or enrichment) of loss-of-function variants in gnomAD compared to a null mutational model. The minimum value is 0, but there is theoretically no maximum value; genes with smaller values (closer to zero) are more intolerant of mutation. pHaplo: Predicted probability of haploinsufficiency. This metric reflects the probability of haploinsufficiency predicted from analysis of large copy-number variants ascertained by microarrays in 950,278 individuals. pHaplo scores ≥ 0.86 indicate that the average effect sizes of deletions are as strong as the loss of function of genes known to be constrained against protein-truncating variants (odds ratio ≥ 2.7). pHaplo scores ≥ 0.55 indicates an odds ratio ≥ 2. ClinGen Dosage sensitivity rate: evidence supporting/refuting the haploinsufficiency and triplosensitivity of genes and genomic regions (https://clinicalgenome.org/). Rate 3/2/1/0: Sufficient/Some evidence/Little evidence/No evidence—for dosage sensitivity. Expression patterns for each gene were also considered by accessing the GTEx portal (https://gtexportal.org/).

## Data Availability

Data sharing is not applicable to this article as no datasets were generated or analyzed during the current study.

## Supplementary Materials

The following supporting information can be downloaded at: https://www.mdpi.com/article/10.3390/genes17010104/s1, Table S1: Genetic findings observed in chromosome 12p. Breakpoints all refer to the Human Genome Assembly GRCh37/hg19. All the DECIPHER patients are indicated by #. -: none; Table S2: Human Phenotype Ontology (HPO) codes useful to identify 12p interstitial deletions clinical symptoms.
