# Supplementary material for: Interstitial 12p Deletion Syndrome: Revised Minimal Critical Region and Review of the Literature
Source: Genes (Basel). 2026 Jan 19;17(1):104. doi: 10.3390/genes17010104 (PMC12840693; doi:10.3390/genes17010104)
Supplement: Supplementary file 1 [file genes-17-00104-s001.zip › Supplementary, Table 1S. Genetic findings observed in chromosome 12p deletions. (1).pdf]

Supplementary, Table S1. Genetic findings observed in chromosome 12p. Breakpoints all refer to the Human Genome Assembly GRCh37/hg19. All the DECIPHER patients are indicated by #. -: none.

**Cases**	**Molecular and Cytogenetic Testing**	**Genetic Results (Start and Stop of the Deletions)**	**Location and Size of Deletions**	**Other Molecular Findings**	**Inheritance of 12p deletions**	**Morbid Genes fully deleted**	**Morbid Genes partially deleted**
Patient 1	A-CGH (8x60K); WES	27,573,443_34,345,585	12p11.23p11.1; 6.77 Mb	-	*de novo* (confirmed)	*DDX11, DENND5B, DNM1L, FGD4, IPO8, PKP2, PPFIBP1, PTHLH, YARS2*	-
Hong- Yong L., et al. [1]	A-CGH (OaCGH44k)	20,724,852_32,201,554	12p12.2p11.21; 11.7 Mb	-	*de novo* (confirmed)	*ABCC9, BHLHE41, ITPR2, DDX11, DENND5B, GYS2, IPO8, KRAS, PPFIBP1, PTHLH, PYROXD1, RECQL, SLCO1B1, SLCO1B3, SOX5*	*PDE3A*
Glaser B., et al. [2]	Karyotype; FISH (BAC clones)	46,XY,del(12)(p12.3p12.1); deletion flanked by BACs RP11-174G6/ RP11-325D10	12p12.3p12.1; 11.68 Mb	-	*de novo* (confirmed)	*ABCC9, ART4, BHLHE41, EPS8, GYS2, KRAS, LDHB, MGP, PDE3A, PDE6H, PLCZ1, PTPRO, PYROXD1, RECQL, SLCO1B1, SLCO1B3, SOX5, WBP1*	*GUCY2C, ITPR2*
Hoppe A., et al. [3]	A-CGH (4x180Kb)	20,951,394_30,124,044	12p12.2p11.22; 9.2 Mb	-	*de novo* (confirmed)	*ABCC9, BHLHE41, ITPR2, GYS2, KRAS, LDHB, PPFIBP1, PTHLH, PYROXD1, RECQL, SLCO1B1, SLCO1B3, SOX5*	-
Huang J., et al. [4]	A-CGH (1x1M)	25,475,995_28,536,312	12p12.1p11.22; 3.06 Mb	arr[GRCh37] 2p22.3(33,955,377_33,980,856)x1,4q34.3 (177,643,308_177,769,701)x3,22q13.31(47,048,713_47,078,331)x1	Inherited from affected parent	*BHLHE41, ITPR2, PPFIBP1, PTHLH*	-
Fryns JP., et al. [5]	Karyotype	46,XX,del(12)(pter----p13.1::p11.2----cen----qter)	±13.7 Mb	-	*de novo* (confirmed)	*ABCC9, APOLD1, ART4, BHLHE41, CDKN1B, EPS8,GRIN2B, GUCY2C, GYS2, ITPR2, KRAS, LDHB, MGP, PDE3A, PDE6H, PLCZ1, PTPRO, PYROXD1, RECQL, SLCO1B1, SLCO1B3, SOX5, WBP11*	-

Soysal Y., et al. [6]	Karyotype; A-CGH (4x180Kb)	46,XX,del(12)(p12.1p11.1); 12p12.1p11.1(24,186,618_34,652,244)x1	12p12.1p11.1; 10.46 Mb	arr[GRCh37] 2p16.3(50,412,234_50,603,279)x1	*de novo* (confirmed)	*BHLHE41, DDX11, DNM1L, DENND5B, FGD4, IPO8, ITPR2, KRAS, PKP2, PPFIBP1, PTHLH, YARS2*	*SOX5*
Nagai T., et al. [7]	Karyotype	46,XY,del(12)(p12.2p11.21)	12p12.2p11.21; 13.3 Mb	-	*de novo* (confirmed)	*ABCC9, BHLHE41, DDX11, DENND5B, DNM1L, FGD4, GYS2, IPO8, ITPR2, KRAS, LDHB, PDE3A, PKP2, PPFIBP1, PTHLH, PIROXYD1, RECQL, SLCO1B1, SLCO1B3, SOX5, YARS2*	-
Stumm M., et al. [8]	Karyotype; A-CGH (1x1M)	10,953,689_30,849,323; Deletion from BAC clone RP11-77I22 to RP11-144O23	12p13.1p11.21; ±19.9 Mb	-	*de novo* (confirmed)	*ABCC9, APOLD1, ART4, BHLHE41, CDKN1B, DENND5B, EPS8,GRIN2B, GUCY2C, GYS2, IPO8, ITPR2, KRAS, LDHB, MGP, PDE3A, PDE6H, PLCZ1, PPFIBP1, PTHLH, PTPRO, PIROXYD1, RECQL, SLCO1B1, SLCO1B3, SOX5, WBP11*	-
Tenconi R., et al. [9]	Karyotype	46,XY,del(12)(p13p11)	12p13p11; ± 30 Mb	-	*de novo* (confirmed)	*A2ML1, AICDA, AKAP3, ABCC9, APOLD1, ART4, ATN1, BHLHE41, CCND2, CD27, CD4, CDKN1B, EPS8,GRIN2B, GUCY2C, GYS2, IPO8, ITPR2, KRAS, LDHB, MGP, PDE3A, PDE6H, PLCZ1, PPFIBP1, PTHLH, PTPRO, PIROXYD1, RECQL, SLCO1B1, SLCO1B3, SOX5*	-
#272344	SNP-array (Affymetrix Cytoscan HD, 750K); FISH	21,296,706_29,349,651	12p12.2p11.22; 8.05 Mb	arr[GRCh37] Xq27.1q27.3(138,650,516_144,234,502)x3	Apparently *de novo;* fourth child and isolated case in the family, which is made up of healthy parents and healthy three siblings; segregation analysis was never performed	*ABCC9, BHLHE41, GYS2, ITPR2, KRAS, LDHB, PPFIBP1, PTHLH, PYROXD1, RECQL, SOX5.*	*SLCO1B1*
#284660	A-CGH (resolution n.a)	22,597,708_29,686,819	12p12.1p11.22; 7.09 Mb	-	*de novo* (unconfirmed)	*BHLHE41, ITPR2, KRAS, PPFIBP1, PTHLH, SOX5*	-

#283599	A-CGH (resolution n.a)	23,155,220_29,617,642	12p12.1p11.22; 6.46 Mb	-	*de novo* (unconfirmed)	*BHLHE41, ITPR2, KRAS, PPFIBP1, PTHLH, SOX5*	-
#263616	A-CGH (resolution n.a)	21,898,057_22,685,048; 25,685,153-29,526783	12p12.1, 787 Kb; 12p12.1p11.22, 3.84 Mb	-	*de novo* (unconfirmed)	*ABCC9, LDHB, BHLHE41, ITPR2, PPFIBP1, PTHLH*	-
#392769	A-CGH (resolution n.a)	21,308,733_34,856,694	12p12.1p11.1; 13.55 Mb	-	*de novo* (unconfirmed)	*ABCC9, BHLHE41, DDX11, DENND5B, DNM1L, FGD4, IPO8, GYS2, ITPR2, KRAS, LDHB, PPFIBP1, PTHLH, PYROXD1, RECQL, SOX5, YARS2*	*SLCO1B1*
#395971	A-CGH (resolution n.a)	20,008,733_33,308,733	12p12.2p11.1; 13.3 Mb	-	*de novo* (unconfirmed)	*ABCC9, BHLHE41, DDX11, DENND5B, DNM1L, FGD4, IPO8, GYS2, ITPR2, KRAS, LDHB, PPFIBP1, PDE3A, PKP2, PTHLH, PYROXD1, RECQL, SLCO1B1, SOX5, SLCO1B3, YARS2*	-
#400822	A-CGH (resolution n.a)	14,808,733_33,308,733	12p12.3p11.1; 18.5 Mb	-	*de novo* (unconfirmed)	*ABCC9, BHLHE41, DDX11, DENND5B, DNM1L, EPS8, FGD4, GYS2, IPO8, ITPR2, KRAS, LDHB, MGP, PDE3A, PDE6H, PLCZ1, PKP2, PTHLH, PYROXD1, RECQL, PTPRO, SLCO1B1, SOX5, SLCO1B3, YARS2*	*GUCY2C*
#287369	A-CGH (resolution n.a)	25,775,746_30,567,013	12p12.1p11.22; 4.79 Mb	arr[GRCh37] 7q11.23(72,643,724_74,142,342)x3	Maternally inherited, both	*BHLHE41, ITPR2, PPFIBP1, PTHLH*	-
#434796	A-CGH (resolution n.a)	23,759,656_31,098,240	12p12.1p11.21; 7.33 Mb	-	*de novo* (unconfirmed)	*BHLHE41, IPO8, ITPR2, KRAS, PTHLH, PYROXD1*	*SOX5*
#503143	A-CGH (resolution n.a)	19,592,050_31,477,869	12p12.3p11.21; 11.89 Mb	-	*de novo* (unconfirmed)	*ABCC9, BHLHE41, DDX11, GYS2, IPO8, ITPR2, KRAS, LDHB, PDE3A,PPFIBP1, PTHLH, PYROXD1, RECQL, SLCO1B1, SOX5, SLCO1B3*	-
#534262	A-CGH (resolution n.a)	25,680,718_28,732,800	12p12.1p11.22; 3.05 Mb	-	*de novo* (unconfirmed)	*BHLHE41, ITPR2, PPFIBP1, PTHLH*	-

#520968	A-CGH (resolution n.a)	16,349,531_27,503,020	12p12.3p11.23; 11.15 Mb	-	*de novo* (unconfirmed)	*ABCC9, BHLHE41, GYS2, ITPR2, KRAS, LDHB, PDE3A, PLCZ1, PYROXD1, RECQL, SLCO1B1, SLCO1B3, SOX5*	-
