# Supplementary material for: Interstitial 12p Deletion Syndrome: Revised Minimal Critical Region and Review of the Literature
Source: Genes (Basel). 2026 Jan 19;17(1):104. doi: 10.3390/genes17010104 (PMC12840693; doi:10.3390/genes17010104)
Supplement: Supplementary file 1 [file genes-17-00104-s001.zip › Supplementary, Table 2S- HPO codes for 12p deletion syndrome.pdf]

Supplementary, Table S2. Human Phenotype Ontology (HPO) codes useful to identify 12p interstitial deletions clinical symptoms.

HPO code	Main Phenotype	Sub Phenotype
HP:0001999	Abnormal facial shape	
HP:0000276		Long face
HP:0002007		Frontal bossing
HP:0000316		Hypertelorism
HP:0000431		Wide nasal bridge
HP:0004322	Short stature	
HP:0002374	Diminished movement	
HP:0001290	Generalized hypotonia	
HP:0001263	Global developmental delay	
HP:0000717	Autism	
HP:0007018	Attention deficit hyperactivity disorder	
HP:0001249	Intellectual disability	
HP:0002463	Language impairment	
HP:0000750		Delayed speech and language development
HP:0001263	Global developmental delay	
HP:0012747	Abnormal brainstem MRI signal intensity	
HP:0012747	Abnormal brainstem MRI signal intensity	
HP:0000648	Optic atrophy	
HP:0000486		Strabismus
HP:0001156	Brachydactyly	
HP:0005863		Type E brachydactyly
HP:0011297		Abnormal digit morphology
HP:0000689	Dental malocclusion	
HP:0000678		Dental crowding
HP:0001626	Abnormality of the cardiovascular system	
HP:0000924	Abnormality of the skeletal system	
HP:0000927		Abnormality of skeletal maturation
